# Prevalence of Dog Erythrocyte Antigens 1, 4, and 7 in Podenco Ibicenco (Ibizan Hounds) from Ibiza Island

**DOI:** 10.1155/2016/1048257

**Published:** 2016-02-29

**Authors:** Eva Spada, Daniela Proverbio, Luis Miguel Viñals Flórez, Blanca Serra Gómez de la Serna, Maria del Rosario Perlado Chamizo, Luciana Baggiani, Roberta Perego

**Affiliations:** ^1^Veterinary Transfusion Unit (REV), Department of Health, Animal Science and Food Safety (VESPA), University of Milan, Via G. Celoria 10, 20133 Milan, Italy; ^2^Centro de Transfusión Veterinario (CTV), Arturo Soria 267, 28033 Madrid, Spain; ^3^Clinical Veterinary Hospital, CEU Cardenal Herrera University, Alfara del Patriarca, 46115 Valencia, Spain; ^4^Laboratorio de Análisis Clínico, Hospital Clínico Veterinario, Universidad Alfonso X el Sabio, Avenida de la Universidad, Villanueva de la Cañada, 28691 Madrid, Spain

## Abstract

The aims of this study were to evaluate the prevalence of Dog Erythrocyte Antigens (DEA) 1, 4, and 7 in Ibizan hounds, to compare the results with the prevalence of DEA in Spanish greyhounds, and to determine the risk of sensitization following the first transfusion of blood not typed for DEA 1 and the probability of an acute hemolytic reaction following a second incompatible transfusion using untyped DEA 1 blood. DEA 1, 4, and 7 status was determined in 92 Ibizan hounds. Results were compared with the previously reported prevalence in Spanish greyhounds. The risks of sensitization and of a hemolytic transfusion reaction were determined amongst Ibizan hounds and between Ibizan hounds and Spanish greyhounds. The prevalence of DEA 1, 4, and 7 was 75%, 98.9%, and 25%, respectively. There was a significantly higher expression of DEA 1 and 7 in Ibizan hounds than in Spanish greyhounds. The probability of sensitization of a recipient dog to DEA 1 with transfusions amongst Ibizan hounds was 18.5% and between Ibizan hounds and Spanish greyhounds was 13.7%. The probability of an acute hemolytic reaction in each group was 3.5% and 1.9%, respectively. There is a higher prevalence of DEA 1 and 7 in Ibizan hounds than in other sighthounds.

## 1. Introduction

There is international standardization of seven canine blood groups as categorized by presence of Dog Erythrocyte Antigen (DEA) 1, 3, 4, 5, 6, 7, and 8 [1]. Other antigen systems have been reported, such as the recently described Dal blood type [2], but none of these systems have been standardized. A donor may express more than one blood group and canine red blood cells can be negative or positive for any given blood type [3].

DEA 1 and 7 are the most important blood types with regard to canine blood transfusions. DEA 1 antigen has recently been classified as a unique DEA epitope with variable surface expression (rather than the expression of different alleles, DEA 1.1, DEA 1.2, and DEA 1.3, as is previously thought) [4] and is present in approximately half the canine population [5–11]. There are no naturally occurring antibodies to DEA 1 in dogs. DEA 1 negative dogs exposed to DEA 1 positive RBCs will become “sensitized” within 9 days by production of anti-DEA 1 antibodies [12, 13]. Anti-DEA 1 antibodies have been reported to cause acute hemolytic transfusion reactions in previously sensitized DEA 1 negative dogs [14].

DEA 7 antigens are reported to occur naturally in between 40 and 72% of the canine population [8, 9, 13, 15, 16]. Between 10 and 40% of dogs negative for these antigens have naturally occurring antibodies to DEA 7 [3, 17] and delayed RBC survival is thought to occur in antigen negative dogs receiving DEA 7 positive blood [15].

There is also a high prevalence of DEA 4 antigens (98–100%) in the canine population [3, 8, 9, 16] and a hemolytic transfusion reaction due to DEA 4 alloantibodies has been reported in a dog [18].

An ideal blood donor does not have blood antigens of types that commonly cause reactions in unmatched recipients. There is no universally agreed definition of a universal canine donor. The most restrictive definition of the universal canine donor would be a dog negative for DEA 1, DEA 3, DEA 5, and DEA 7 and positive for DEA 4. Since 98% of dogs are positive for DEA 4, the rarity of DEA 4 negative dogs means that this antigen is unlikely to influence donor selection. Some transfusion specialists do not exclude DEA 7 positive dogs from the donor pool. In fact the concept of the universal donor in dogs has long been debated since the tests for DEA 3 and DEA 5 antigens are often not available and the quality of these tests is variable; currently typing sera are available only for DEA 1, 4, and 7 antigens. As previously proposed [14], universal donors are therefore often considered to be dogs that are DEA 1 and 7 negative. Point-of-care tests are currently only available for DEA 1, although, in specialized laboratories, polyclonal antisera are available for DEA 4 and DEA 7 typing.

Ibizan hounds are medium-size sighthounds originating from the island of Ibiza. They are traditionally used in the Balearic Islands (and less so in Spain and France) to hunt rabbits and other small game. In recent times this breed has been disseminated worldwide through a variety of adoption programs. The Ibizan hound is classified by the Fédération Cynologique Internationale (FCI) in Group 5 (spitz and primitive types) and in section primitive type-hunting dogs. This dog is a typical and robust representative of one of the oldest breeds still in existence (http://www.fci.be/en/nomenclature/IBIZAN-PODENCO-89.html).

The aims of this study were threefold: (1) to evaluate the prevalence of DEA 1, DEA 4, and DEA 7 blood type in Ibizan hounds; (2) to compare the results with the previously published prevalence of DEAs in other sighthounds (i.e., Spanish greyhound); (3) to determine the risk of recipient sensitization following the first transfusion of blood not typed for DEA 1 and the probability an acute hemolytic reaction following a second incompatible transfusion of blood untyped for DEA 1 both amongst Ibizan hounds and between Ibizan hounds and Spanish greyhounds.

## 2. Material and Methods

### 2.1. Samples

In this prospective study EDTA-anticoagulated blood samples were collected from 92 healthy owned Ibizan hounds in January 2015. Dogs were between 1-year and 3-year old, 89 were female (96.7%) and 3 were male (3.3%), and all were living on Ibiza Island, Spain.

With the owners' consent cephalic blood samples were collected with a 23 G needle (Sterican Braun®, B. Braun Melsungen AG, Melsungen, Germany) connected to a 2 mL syringe (Injekt Braun®, B. Braun Melsungen AG, Melsungen, Germany). They were transferred to a 1 mL EDTA-anticoagulated tube and were stored between 4 and 5°C. All blood samples were collected as part of a study to determine the distribution of blood groups in this breed. This study was conducted according to European legislation (2010/63/EU). In all samples blood typing was performed as described below.

### 2.2. DEA 1 Blood Typing

DEA 1 status was determined using a commercially available card agglutination technique (RapidVet-H, Canine DEA 1.1, Agrolabo SpA, Scarmagno, Turin, Italy) according to the manufacturer's instructions and as previously described [19, 20]. The principle of this card-based agglutination test is a visible hemagglutination reaction resulting from the binding of the DEA 1 RBC surface antigen to a murine monoclonal antibody that is lyophilized on the card test.

### 2.3. DEA 4 and DEA 7 Blood Typing

Analysis for DEA 4 and 7 antigens was performed by gel column agglutination within microtubes (ID-CARD NaCl, Enzyme Test and Cold Agglutinins, DiaMed, Cressier FR, Switzerland) as previously described [21] using polyclonal anti-DEA antibodies produced by Animal Blood Resources International (ABRINT, Stockbridge, MI, USA).

Anti-DEA 4 and 7 antibodies were imported and used in this study with the authorization of Italian Health Minister (protocol authorization number 0021278-15/10/2014-DGSAF-DGSAF-P). Blood-typing was performed at the Veterinary Transfusion Unit (REV) of the University of Milan, Milan, Italy.

Briefly 25 *μ*L of a 0.8% RBC suspension made by suspending 10 *μ*L of the RBC pellet in 1 mL of low ionic strength solution (LISS, ID-DILUENT 2, modified LISS solution, DiaMed, Cressier FR, Switzerland) was mixed with 25 *μ*L of DEA 7 antibodies or with 15 *μ*L of DEA 4 antibodies in the reaction chamber of saline gel columns. For all samples a negative control column with saline solution was included. The gel columns were incubated at 4°C for 30 minutes and were then centrifuged in a special gel column card centrifuge (ID-CENTRIFUGE 24 S, DiaMed-ID Micro Typing System, Cressier sur Morat, Switzerland) at 80 × g for 10 minutes. Finally the gel column cards were evaluated for presence and strength of agglutination. Only results validated by negative controls were included in analysis.

The cards were visually interpreted as follows: (0) negative, when all RBCs were at the bottom of the column; 1+, when very few RBC agglutinates were dispersed in the lower part of the gel, with most RBCs at the bottom of the tube; 2+, when all RBCs were agglutinated and dispersed in the gel; 3+, when some RBC agglutinates were dispersed in the upper part of the gel and most of the RBCs formed a red line on the surface of the gel; and 4+, when all RBCs formed a red line on top of the gel. Results were interpreted as negative if no agglutination or 1+ agglutination was present, whereas ≥2+ agglutination reactions were considered positive.

### 2.4. Sensitization and Transfusion Risk Analysis for DEA 1

The probability of an Ibizan hound becoming sensitized following the first transfusion of blood that was neither cross-matched nor typed for DEA 1 was calculated using the following formula [5, 10]:

(1) $$\begin{array}{c} \left[\frac{\left(\%\text{ DEA 1 negative}\times \%\text{ DEA 1 positive}\right)}{100}\right]. \end{array}$$

The probability of the same dog developing an acute hemolytic reaction with a second incompatible transfusion using untyped blood from any other dog was calculated using the following formula [10]:

(2) $$\begin{array}{c} \frac{\left[\left(\%\text{ DEA 1 negative}\times \%\text{ DEA 1 positive}\right)\times \%\text{ sensitization for the first transfusion}\right]}{10,000}. \end{array}$$

### 2.5. Statistical Analysis

Results were analysed by absolute prevalence analysis. Using contingency tables or Fisher's exact test the prevalence of DEA 1, 4, and 7 and universal donors in Ibizan hounds calculated in this study was compared with a population of 205 Spanish greyhounds (galgo) in which the prevalence of DEA 1, 4, and 7 and universal donors (i.e., DEA 1 and 7 negative and DEA 4 positive) was 54.6%, 100%, 8%, and 46.7%, respectively [22].

All statistical analysis was performed using statistical software (Medcalc software version 14.10.2, Mariakerke, Belgium) with significance set at *P* < 0.05.

## 3. Results

### 3.1. DEA 1, DEA 4, and DEA 7 Prevalence

For DEA 1 blood type, 69 (75%) dogs tested positive and 23 (25%) tested negative. For DEA 4 blood type, 91 (98.9%) dogs tested positive (all showed 4+ agglutination) and 1 (1.1%) dog tested negative. For DEA 7 blood type, 23 (25%) dogs tested positive (16 samples showed 3+ agglutination and 7 samples showed 2+ agglutination) and 69 (75%) were negative (no samples showed agglutination). Of the 92 dogs, 16 (17.4%) were DEA 4 positive only (universal donors) and 1 (1.1%) dog was negative for all DEA. All control samples in the gel columns tested negative (absence of agglutination).

### 3.2. Comparison of DEA 1, 4, and 7 Prevalence with Spanish Greyhounds

Ibizan hounds had a significantly higher prevalence of DEA 1 and DEA 7 positivity than Spanish greyhounds (*P* = 0.005 and *P* = 0.002, resp.). The prevalence of universal donors (DEA 1 and DEA 7 negative and DEA 4 positive or negative) was lower in Ibizan hounds (*P* = 0.000008) than in Spanish greyhounds. There was no significant difference in DEA 4 prevalence between the 2 breeds.

### 3.3. Risk of Sensitization for DEA 1 and a Hemolytic Acute Reaction in an Untyped Recipient

The probability of an Ibizan hound recipient becoming sensitized following the first transfusion of blood from an Ibizan hound donor that was not cross-matched nor typed for DEA 1 was approximately 1 in 5 (18.8%). The probability of the same dog developing an acute hemolytic reaction with a second incompatible transfusion using blood untyped for DEA 1 from any other Ibizan hound was 3.5%.

The probability of a recipient Ibizan hound becoming sensitized following the first transfusion of blood from a Spanish greyhound that was not cross-matched nor typed for DEA 1 was approximately 1 in 7 (13.7%). The probability of the same dog developing an acute hemolytic reaction with a second incompatible transfusion using untyped DEA 1 blood from Spanish greyhound was 1.9%.

## 4. Discussion

There are breed and geographical differences in the prevalence of different blood group antigens [8, 15]. It is essential to type donor blood in order to select compatible canine blood donors. The prevalence of positivity to antigens in a potential donor population can be used to calculate the likelihood that such an antigen will cause an adverse transfusion reaction and therefore the risk associated with any transfusion can be assessed.

In this study the prevalence of DEA 1 expression in Ibizan hounds was 75%, which is higher than previously reported in other pure breeds [7–9, 15, 16] and in cross-breed dogs [6]. The prevalence of DEA 1 in Ibizan hounds was also much higher than previously reported in other sighthounds, such as Greyhounds and Spanish greyhounds, in which the prevalence of DEA 1 has been reported to be 13.1% [16] and 51.7% [11], respectively. The prevalence of DEA 1 was statistically significantly higher in Ibizan hounds than in a population of Spanish greyhounds tested for DEA 1 [22].

All 92 Ibizan hounds were blood typed and only one was DEA 4 negative. This prevalence of DEA 4 expression (98.9%) is in agreement with the prevalence in the general canine population [6, 7, 9, 16, 21].

The prevalence of DEA 7-positive dogs in the study population (25%) was similar to that reported in Golden Retrievers in USA and Brazil (25% and 27% resp.) [7, 9] but higher than the prevalence reported in cross-breeds (11%) and German Shepherd dogs (8%) in Brazil [6] and lower than in Greyhounds in USA (29.1%) [16] and Turkish Kangal dogs (71.1%) [8]. The prevalence of DEA 7 expression in Ibizan hounds was statistically significantly higher than in a population of Spanish greyhounds previously tested by the authors for DEA 7 [22]. These results confirm that as for other canine blood types, the prevalence of DEA 7 differs between populations.

The prevalence of universal or “ideal” donors, that is, dogs positive only for DEA 4 or negative for all DEA, in our population of Ibizan hounds, was 18.5%. This is lower than the prevalence of 57.3% found in greyhounds [16] and in a population of Spanish greyhounds previously blood typed, in which prevalence of universal donors was 46.7% [22].

In this study the probability of a recipient becoming sensitized and produced antibodies against DEA 1 following the first transfusion of blood that was neither cross-matched nor typed for DEA 1 was 18.8% (1 in 5) amongst Ibizan hounds and 13.7% (1 in 7) between Ibizan hounds and Spanish Greyhounds. These probabilities were lower than previously reported in dogs from Portugal [10], in Spanish greyhounds in Spain [11] and in dogs in Brazil [5] in which probabilities were 24.5%, 22.9%, and 24.9%, respectively. The risk of a hemolytic transfusion reaction was also lower than the 6% reported in a previous study [10] since there was a higher prevalence of DEA 1 positivity in the Ibizan hounds (so these dogs could receive either DEA 1 negative or DEA 1 positive blood at first transfusion without transfusion reactions).

The gel agglutination technique has been used for decades. This test is sensitive for the detection of DEA 1 and is suited for screening blood donors in a blood bank program [19, 20]. This study used, for only the third time in the canine blood typing, column gel agglutination with polyclonal antibodies for DEA 4 and DEA 7 [21, 22]. The column gel agglutination test is simple to perform, does not require washing of RBC, requires only small sample and reagent volumes, and yields results that are simple to read and stable over time (for up to three days). The study that validated this technique with polyclonal antibodies for DEA 4 and 7 demonstrated that the test was not 100% sensitive for identification of DEA 7 [21]. In fact, when gel agglutination was compared with tube agglutination (considered the gold standard), there were 12 discordant results for DEA 7 (concordance of 84%). The gel agglutination test had a specificity of 100% and a sensitivity of 53% for identification of the DEA 7 positive samples when compared with tube agglutination [21]. This may represent a limitation of our study as the true prevalence of DEA 7 positive dogs may have been higher than detected using this test.

Another limitation of this study was that the study population of Ibizan hounds was almost exclusively female, mirroring the environment in which Ibizan hounds live. Hunters run these dogs in mostly female packs, as the female is considered the better hunter. In addition we did not know how closely related the dogs used in this study were. It is possible that a closed population of closely related individuals could bias the prevalence of blood types. Finally DEA 3, DEA 5, and Dal blood types were not tested since relevant antisera were unavailable at the time the study was performed.

## 5. Conclusion

The population of Ibizan hounds studied here showed a different prevalence for DEA 1 and 7 with respect to previous reports of other sighthounds. Although the risk of sensitization and an acute transfusion reaction following incompatible blood transfusion is low, it remains best practice to blood type and cross-match recipients before transfusion.
